# Transcriptome sequencing reveals e-cigarette vapor and mainstream-smoke from tobacco cigarettes activate different gene expression profiles in human bronchial epithelial cells

**DOI:** 10.1038/srep23984

**Published:** 2016-04-04

**Authors:** Yifei Shen, Michael J. Wolkowicz, Tatyana Kotova, Lonjiang Fan, Michael P. Timko

**Affiliations:** 1 Research Center for Air Pollution and Health and Institute of Crop Science, Zhejiang University, Hangzhou 310058, China; 2 Department of Biology, University of Virginia, Charlottesville, VA 22903, USA; 3 Department of Public Health Sciences, University of Virginia, Charlottesville, VA 22903, USA.

## Abstract

Electronic cigarettes (e-cigarettes) generate an aerosol vapor (e-vapor) thought to represent a less risky alternative to main stream smoke (MSS) of conventional tobacco cigarettes. RNA-seq analysis was used to examine the transcriptomes of differentiated human bronchial epithelial (HBE) cells exposed to air, MSS from 1R5F tobacco reference cigarettes, and e-vapor with and without added nicotine in an *in vitro* air-liquid interface model for cellular exposure. Our results indicate that while e-vapor does not elicit many of the cell toxicity responses observed in MSS-exposed HBE cells, e-vapor exposure is not benign, but elicits discrete transcriptomic signatures with and without added nicotine. Among the cellular pathways with the most significantly enriched gene expression following e-vapor exposure are the phospholipid and fatty acid triacylglycerol metabolism pathways. Our data suggest that alterations in cellular glycerophopholipid biosynthesis are an important consequences of e-vapor exposure. Moreover, the presence of nicotine in e-vapor elicits a cellular response distinct from e-vapor alone including alterations of cytochrome P450 function, retinoid metabolism, and nicotine catabolism. These studies establish a baseline for future analysis of e-vapor and e-vapor additives that will better inform the FDA and other governmental bodies in discussions of the risks and future regulation of these products.

Cigarette smoking results in injury to the epithelial cells of the human respiratory tract^1^ and has been implicated as a causative factor in the development of chronic obstructive pulmonary disease (COPD) and lung cancers^2^. COPD is a major cause of chronic morbidity and mortality worldwide^3^,^4^ and is the second leading cause of death in the US^5^,^6^,^7^. In addition to its effects on lungs^8^ inhalation of tobacco smoke also causes damage and disease to other tissues and organ systems in the body, including of the oral cavity, pharynx,-larynx-esophagus, digestive and urinary tracts, and pancreas^9^,^10^.

Mainstream smoke (MSS) resulting from the combustion of tobacco cigarettes is a mixture of over 6,000 individual chemical constituents in both gas and particulate phases^10^,^11^,^12^,^13^. In this mixture, nicotine and its derivatives are known pharmacologically-active components. Although nicotine itself plays a minor role in the causation of smoking-induced diseases, it has been consistently linked to smoking addiction because of its ability to stimulate release of the pleasure reward neurotransmitter dopamine in the brain^14^ thought to be an important positive reinforcement in continued smoking. Nicotine is readily released from its receptor and rapidly metabolized and consequently for some individuals the reduction or elimination of nicotine consumption via smoking is difficult.

While the direct effects of low doses of nicotine on cellular function appear to be largely benign the repetitive exposure to the multitude of other cytotoxic components in MSS results in smoking-related cellular damage and disease, a fact well-documented for decades^15^. Only recently has intensified societal awareness of the hazards of tobacco consumption resulted in the enactment of federal legislation to significantly to reduce the health burden resulting from the harmful effects of smoking through the US Family Smoking Prevention and Tobacco Control Act of 2009^16^,^17^. These strident regulations seek to control current and future tobacco products and promote the use of modified-risk tobacco products (MRTPs) for those unable or unwilling to stop smoking^16^,^17^. MRTP products are thought to have demonstrated reduction of harm and risk of tobacco smoking-related disease compared to mainstream smoke (MSS) generated from conventional tobacco cigarettes^18^,^19^,^20^.

Among MRTPs on the market, electronic nicotine delivery systems (ENDS), popularly known as electronic cigarettes (e-cigarettes) have become increasingly popular in the US since their introduction in 2007^21^,^22^. E-cigarettes outwardly resemble conventional cigarettes and typically consist of a battery (either disposable or rechargeable), a reservoir containing a liquid mixture typically composed of propylene glycol, glycerol, nicotine, flavorants, and other additives, and a heating element linked to an air flow activated sensor such that upon puffing the atomizer generates a warm aerosol mist or e-vapor^23^,^24^.

Exposure of the human respiratory tract to MSS from tobacco cigarettes induces a myriad of effects directly measurable at the cellular and genetic level^25^,^26^. In addition to alterations in cellular structure and metabolism, global changes in gene expression and alterations in small (miRNA) populations have been documented in human lung epithelial cells following exposure to MMS and cigarette smoke condensates using microarray-based gene expression profiling technology^27^,^28^,^29^,^30^,^31^,^32^,^33^ and high-throughput RNA sequence (transcriptome) analysis^34^,^35^,^36^,^37^,^38^. These studies indicate that exposure to tobacco smoke results in rapid and often prolonged activation of gene expression associated with antioxidant and detoxification pathways as well as changes in the expression of genes controlling cell structure, adhesion, cell cycle, immune modulatory, and apoptosis. Several studies have also shown that smoking-induced alterations in human bronchial epithelial (HBE) cell gene expression also varies among individuals particularly in activation/deactivation of genes controlling antioxidant- and drug-metabolizing processes^39^,^40^,^41^.

It has also been reported that HBE cells exposed to equivalent doses of smoke condensate from 10 different brands of cigarettes resulted in altered gene expression that was both generic and product specific reflecting their composition (Pickett *et al*. 2010).These investigators identified 21 genes encoding antioxidant and detoxifying functions that were differentially activated/deactivated two-fold or greater following exposure to condensates of 9/10 of the cigarettes, but that a large number of genes were uniquely affected by the different product specific condensates. They interpreted these findings as indicative that not all cigarettes generate the same toxins or concentration of toxins. Another *in vitro* study of HBE cells exposed to smoke condensate from different brands of cigarettes with similar tar and nicotine content showed that about half of the differential gene expression observed was unique to the individual cigarette brand^42^.

Subsequent studies have also documented that smoking related alterations can be observed the transcriptome of HBE cells from current smokers and individuals reporting never to have smoked^27^ and the nasal, oral, and bronchial epithelial cells in smokers and nonsmokers^8^. Relating gene transcriptional alteration to disease etiology is difficult both in regard to COPD as well as various lung and oral cancers. Clearly additional work is needed to relate both gene structural alterations and epigenetic effects influencing processes and pathways to could affect cellular function and promote oncogenicity.

At the present time, data on the effects of the aerosols (e-vapors) produced by e-cigarettes on human cellular function are limited due in part to the fact that the devices are highly variable in construction, their charging liquids and aerosol vapors produced are highly variable in composition, and while the FDA holds jurisdiction over the product, it remains largely unregulated. Reports from the FDA suggest that e-vapor contains fewer potentially toxic chemicals than MSS from tobacco cigarettes^17^ but still harbors considerable risk primarily in products that contain tobacco extracts, nicotine, and nicotine derivatives^17^,^20^. Consistent with this warning Cressey *et al*.^43^ reported that exposure of immortalized HBE cells to e-liquid is capable of inducing carcinogenicity-related gene expression similar to that reported following exposure to tobacco smoke. Even in the absence of nicotine and other additives and flavorants, e-vapor produces small particles similar to those present in tobacco cigarette smoke that can be deposited in the bronchial cavity. Propylene glycol, the primary component of e-vapor, has been reported to cause acute upper airway irritation in non-smokers^44^. Consistent with this observation is the report by Wu *et al*.^45^ who showed that exposure of HBE cells to nicotine-free e-liquid promotes pro-inflammatory response as evidenced by the induction of the pro-inflammatory cytokine IL-6 and inhibition of expression of SPLUNC1 (short palate, lung, and nasal epithelium clone 1). Additionally, the e-liquid induced inhibition of SPLUNC1 expression in HBE cells reportedly compromised the ability of the cells to mount innate immune response against HRV infections. Kogel *et al*.^46^ compared the response of HBE cells to various doses and duration of exposure to aqueous extracts (AE) prepared from MMS and e-vapor. Analysis of 11 distinct indicators of cellular toxicity performed using a high-content screening and microarray-based whole genome expression analysis revealed that the number of genes differentially activated in response to AE prepared from e-vapor was stunningly low compared to the numbers activated by AE from MMS condensates. These investigators suggested that this reflects an overall reduction in the numbers of cellular pathways activated. Finally, an additional concern with e-cigarettes has been raised by reports of the presence of diethylene glycol, genotoxins, and tobacco specific nitrosamines (e.g., NNN, NNK) known to be carcinogenic in humans in some-liquid preparations^17^,^22^.

The direct effects of e-vapor exposure on global gene expression in HBE cells has not been previously examined. Given the limited information currently available on the effects of “vaping” (e-cigarette use), such an analysis is highly merited in order to fully understand the risk associated with use of these products. To this end, we have used whole transcriptome sequencing (RNA-Seq) to characterize the gene expression alterations associated with exposure e-vapor aerosols either containing or lacking nicotine generated by a commercial e-cigarettes device. We have compared the e-vapor elicited transcriptomic profiles to those of differentiated HBE cells exposed to filtered air controls and MSS generated by combustion of a tobacco reference cigarette. Unlike previous studies of e-cigarette exposure that used primarily exposure to e-liquid, our studies compare e-vapor to MSS under identical experimental conditions. As a result we are able to demonstrate that significant differences in cellular response exist between e-vapor exposed and MSS exposed cells and demonstrate that e-vapor has clear and significant effects on cellular function that underscore the potential risk to e-cigarette use.

## Results

### Establishment of differentiated HBE cells in an *in vitro* air-liquid interface culture

Differentiated human bronchial epithelial (HBE) cell cultures were established from cells taken from two independently obtained healthy non-smoking female donors using previously published methodologies^35^. By 21–23 days post seeding the differentiated primary HBE cells formed a pseudo-stratified mucociliary morphology containing about 50–70% ciliated cells, about 25% goblet cells, and about 30% basal cells (Supplementary Fig. S1). Immuno-histological staining with tubulin and α-MUC5AC antibody confirmed cellular differentiation and allowed quantitation of cell types. As expected, measurements of TEER increased in the cultures as differentiation proceeded indicating that tight junctions were forming between the cells prior to any stress exposure.

### Effects of air, e-vapor, and MSS exposure on transcriptional activities of HBE cells

To study the effects of MMS and e-vapor exposure on the transcriptional activities of differentiated HBE cells, we exposed 21 to 23 day differentiated cells growing in air-liquid interface culture to filtered air (AT, air treated), filtered air diluted mainstream smoke (MSS) generated from the combustion of 1R5F tobacco reference cigarettes (ST, smoke treated), and aerosol (e-vapor) generated from a commercial MRTP (EV, e-vapor treated) for periods of 1 h. The e-vapor contained either no nicotine (0 mg/ml in standard e-liquid provided by the manufacturer) or 16 mg/ml of nicotine (designated EV0 and EV16, respectively, in the text). At the end of the exposure, cells were either collected immediately, flash frozen in liquid nitrogen, or allowed to recover in the presence of filtered air and circulating fresh growth media for periods of 4 h and 24 h. At the 4 h and 24 h recovery times, cells were harvested and flash frozen as with control samples. Approximately 7.5 × 10^4^ cells per insert were seeded on the air/liquid interface cultures with continuous circulating growth media and cell viability was measured before and after exposure to ensure that equivalent cell viability was present in all samples. For comparisons of MMS and e-vapor Total Particulate Matter (TPM), Total Suspended Particulate (TSP), and nicotine delivered to the chamber quantified to ensure approximately equivalent dosing of cells. At the three time points (1 h exposure, 4 h post-exposure, 24 h post-exposure) harvested cells were then used in the preparation of total RNA for subsequent use in Illumina based RNA-Seq analysis.

**Figure 1:**
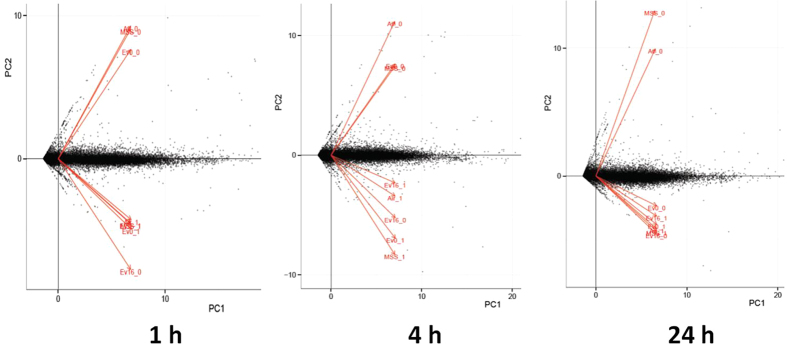
PCA results of transcriptomic data of 24 samples at three exposure time points from HBE cells. In the PCA plots, the samples are divided into two clusters, each cluster represents a donor in the experiment (two donors were termed by *_ 0 and *_ 1, respectively). RNAs were collected from HBE cells exposed to MSS or e-vapor as indicated in the Methods. Based on the PCA results, four samples were deemed to be “outliers” and are excluded in our following analysis.

As shown in Table 1, following whole transcriptome sequencing (RNA-Seq) analysis, we were able to generate about 500 Gb total paired-end RNA-seq data (representing ~20 Gb for each sample) in fastq format. FASTQC^47^ was used to generate quality control (QC) metrics (base quality distribution) for initial reads and low quality read ends were trimmed using Sickle^48^. The sequencing reads obtained from the various samples were combined and mapped to the human reference genome (EnsEMBL, GRC37) using Tophat v2.0.0^49^. Cufflinks was used to assemble the transcripts after the alignment step and all the transcripts generated from the sequence samples were merged by the Cuffmerge. We then applied Principal Component Analysis (PCA) to examine the clustering of the individual samples, to explore the relationship between different samples, and aid in identifying any samples that did not cluster as expected (i.e., outlier samples from their expected sample mates). To find similarity in expression profiles among the different samples, we also applied PCA to gene expression levels. As shown in Fig. 1, the samples were divided into two clusters by two principal components which likely represent the two donors used in our experiment. Such donor specific responses are to be expected. Following this analysis, we determined that four individual samples were outliers and we excluded these samples in any further analysis (Supplementary Fig. S2).

To ensure the reliability our results, we compared the RNA-seq determinations for various genes in three different time points (1 h, 4 h, and 24 h) in the two matched donors. The results of this analysis (Supplementary Fig. S3) indicated that the expression level of the individual genes in donor 1 have a high correlation with the expression levels of the corresponding genes in donor 2. To further test the reproducibility of our data we chose six well documented housekeeping genes^50^ and compared their expression under the various treatments in the two donors. As shown in Supplementary Table S27) the levels of expression of these housekeeping genes were stable in our RNA-seq data further validating the reliability and reproducibility of our methods.

### Effects of air, e-vapor, and MSS exposure on transcriptional activities of HBE cells

#### Differential gene express in air treated (AT) control cells

In our studies, we considered exposure to filtered air as our control condition. Under these conditions we expected that cells growing at the air-liquid interface would exhibit minimal alteration of gene expression following initial exposure of 1 h (AT 1) and during the 4 h and 24 h recovery period (AT 4 and AT 24, respectively). This conclusion is validated (Supplementary Fig. S4) by our comparison of the number and type of differentially expressed genes observed in the AT control samples. A total of 104 and 87 differentially expressed genes (DEGs) were identified in AT 4 and AT 24 samples compared to AT 1, of which 13 were unique to the AT 4 sample. Thus, it appears that only minimal perturbation of the transcriptional activity of the HBE cells is occurring in response to air treatment as would be expected. Of the DEGs identified, DAVID analysis indicated that these are primarily associated with pathways involved with regulation of cell growth (response to growth factors), regulation of cell motion/cell migration, transcriptional control of growth and differentiation, and protease inhibition (Supplementary Tables S1 and S2).

Using AT control as baseline, we next sought to determine how HBE cells respond transcriptionally to exposure to e-vapor (plus (EV16) or minus (EV0) added nicotine) over the same exposure (EV0 1 h, EV16 1 h and ST 1 h) and recovery (EV0 4 h, EV16 4 h; EV0 24 h, EV16 24 h; ST 4 h and ST 24 h) time frame.

#### Differential gene expression following exposure to MSS

**Figure 2:**
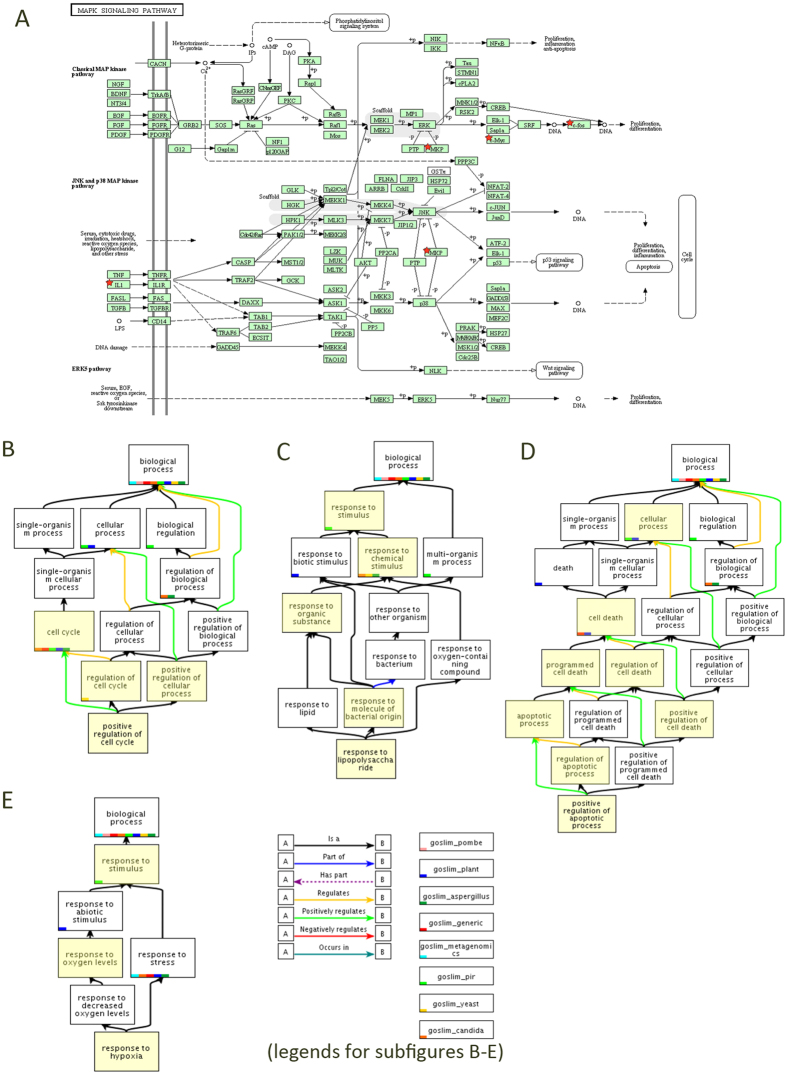
Five significant enriched pathway involved in the response of HBE cells to MSS and e-vapor. (**A**) The MAPK signaling pathway can be detected enriched in MSS 4 h and MSS 24 h treated cultures. (**B**) Cell cycle cluster. (**C**) Apoptosis cluster (**D**) Response to organic substance cluster (**E**) Response to hypoxia cluster. The genes included in this pathway are marked in red stars. Clusters B ~ E can be detected in MSS 1 h, MSS 4 h, MSS 24 h and EV 16 1 h samples. The GO term graphs are from the Gene Ontology database. The GO term which significantly enriched in each cluster is colored in yellow.

We then compared gene expression in HBE cells exposed to MSS using AT control samples as our baseline. A total of 49 genes are significantly (p < 0.05, Benjamini-Hochberg [BH] multiple test correction) differentially expressed in the ST treated HBE cells compared with the AT control at 1 h exposure (Supplementary Fig. S5). Of these genes, 16 are down-regulated (Table 2 and Supplementary Table S3). The relatively high number of down regulated genes suggests that many cellular processes are either inhibited following the toxic insult. Analysis of the nature of these genes indicate that a number of pathways are affected (Fig. 2; Supplementary Table S12) among which signal transduction (Fig. 2A), regulation of cell cycle (Fig. 2B), apoptosis (Fig. 2C), response to organic substances (Fig. 2D) and response to hypoxia (Fig. 2E) are significantly enriched at this time point. Pathways regulated at this time point indicates that the negative regulation of transcription factor which suggested that the toxic compounds of the smoke suppressed the activity of different pathways through transcription factors. The results of the GSEA also supported what we discovered in DAVID. Majority of the enriched pathways which related DNA damage response, such as P53 dependent G1 DNA damage response and P53 dependent/independent G1 S DNA damage checkpoints, were fall in the category of cell cycle. In company with the enrichment of cell cycle category, the category of DNA repair and DNA replication were shown significantly regulated in the mainstream smoke treated culture compared with the air treated control, which means many pathways related with DNA damage repair and cell cycle were responsive to the smoke (Supplementary Table S17).

**Figure 3:**
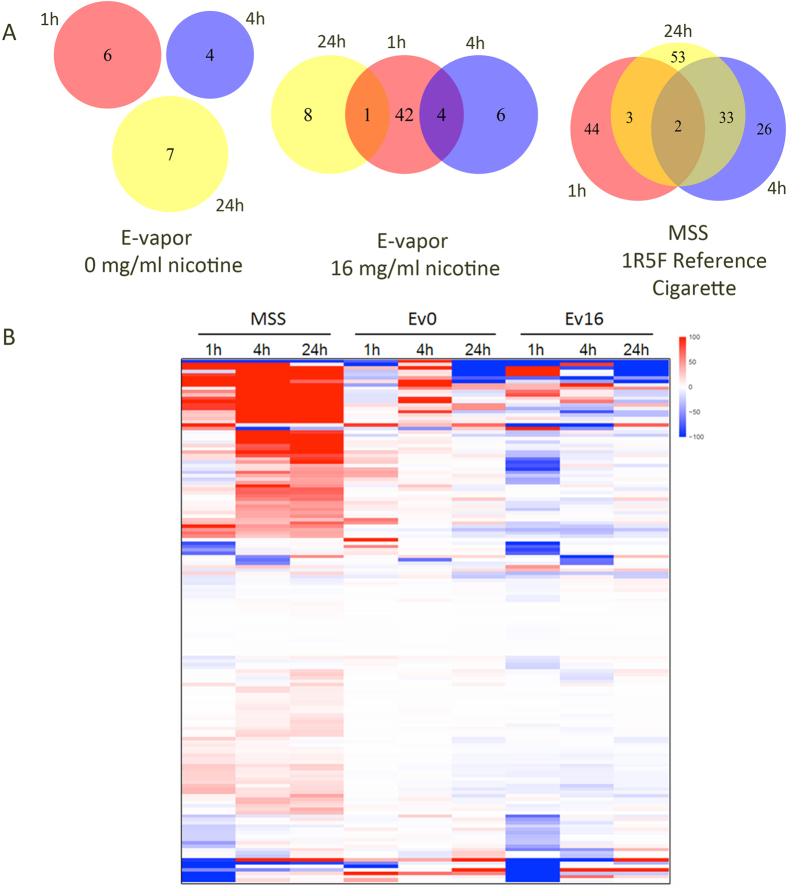
Differentially expressed genes (DEGs) (5% FDR) of HBE cells responsive to the MSS and E-vapor at three post-exposure times. (**A**) Venn diagram of the number of significant regulated genes compared with the air treated (AT) control in three conditions. MSS, mainstream smoke treated, EV0, e-vapor (0 mg/ml nicotine)-treated, EV16, e-vapor (16 mg/ml nicotine)-treated. (**B**) Heatmap of DEGs in the various treatments relative to AT control. Values presented are Fragments per Kilobase of exon model per Million mapped reads (FPKM).

At 4 h, the majority of differentially expressed genes were up-regulated compared with air treated control (95%) (Fig. 3B, Table 2 and Supplementary Table S4). There were significant changes in expression pointing to a renewal of cellular activity. The response to stress and DNA damage stimulus were significantly overrepresented up-regulated categories. The result reveals that the cells attempt to recover from exposure to cigarette smoke. Genes up-regulated in the cell cycle cluster were positive regulator of cell proliferation which indicated that cell cycle overrepresentation was occurring and possibly enabling DNA damage repair or preceding apoptosis. Both of the categories were significantly up-regulated in the smoke-treated culture (Supplementary Table S13). The same results were got from the GSEA. Pathways involved in the category of cell cycle, gene expression were significantly enriched. DNA damage response and regulation of cell cycle, subcategories of cell cycle, were detected to be enriched in the smoke treated cultures at 4 h after exposure (Supplementary Table S18) which is the same as what we found in the 1 h exposure smoke treated samples. Compared with the results of 1 h exposure samples, more pathways were significantly enriched in the category of signaling transduction, including two main subcategories, signaling by *Wnt* and signaling by G-protein coupled receptors (GCPRs) (Supplementary Table S18). *Wnt* signaling regulates cell development and proliferation through β-catenin and reduced *Wnt*/β-catenin signaling activity was observed in the airway epithelium of smokers with or without COPD^51^ and *Wnt* is activated during smoking-induced lung tissue damage and inflammation leading to significantly increased CXCL8/IL-8, IL-6, CCL5/RANTES, CCL2/MCP-1 and vascular endothelial growth factor (VEGF) secretion^52^. The MAPK signaling pathway also can be detected significantly enriched in GSEA, the same as the result of DEGs enrichment analysis (Fig. 2A).

In ST 24 h samples, 88/91 genes deemed significantly differentially expressed were up-regulated (Fig. 3B, Table 2 and Supplementary Table S5). Many of these genes clustered in pathways involved in cell cycle regulation, MAPK signaling, and response to hypoxia (Supplementary Table S14). Since these pathways were also observed to contain significantly up-regulated genes at earlier time points (Supplementary Tables S12 and S13) their persistent up-regulation implies that the toxicity of smoke exposure is sustained long after MMS exposure has stopped. This persistence has been noted previously by others^35^,^53^. The up-regulation of apoptosis in ST 24 h samples suggests that excessive and irreparable DNA damage has occurred in the HBE cells.

The result of our analysis show that there are many more overlapping DEGs in the transcriptomes of HBE cells of ST 4 h and ST 24 h samples (Fig. 2A). In contrast, the DEGs identified in ST 1 h samples were extremely different from the ST 4 h and ST 24 h samples. This is not surprising rapid changes in gene expression patterns would be expected in cells undergoing the effects of MMS exposure whereas during the 4 h and 24 h periods post-exposure readjustment and renewal of cellular activity likely initiate and persist. The results of GSEA reflect this in that indicates many pathways were affected by smoke treated 24 h post-exposure, including. The most significantly enriched pathways in ST 24 treated HBE cells fall in the category of cell cycle regulation, mitotic checkpoint regulation, synthesis of DNA/DNA damage response, regulation of mRNA stability and translation. The key *Wnt* and GCPR signal transduction pathways were still significantly enriched in ST 24 h cultures (having been activated at the ST 4 h time point).

**Figure 4:**
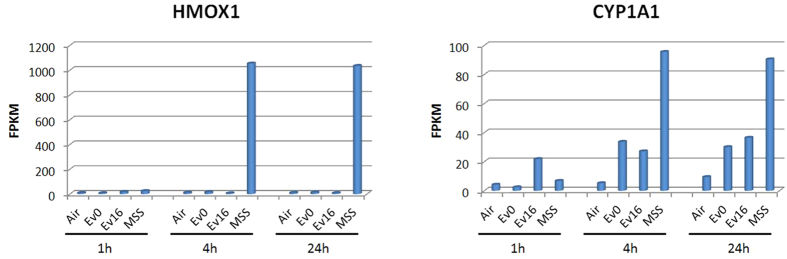
HMOX1 and CYP1A1 in HBE cells under different exposure treatments. Shown are the expression levels of HMOX1 and CYP1A1, two biomarkers of smoke exposure, in HBE cells under different exposure treatments and times. AT, air treated control, MSS, mainstream smoke treated, EV0, e-vapor (0 mg/ml nicotine)-treated, EV16, e-vapor (16 mg/ml nicotine)-treated. Values presented are FPKM.

Some of the largest fold-changes in expression were in genes involved in cell cycle regulation, apoptosis, and response to stimuli, including genes previously implicated in response to smoke exposure, hemoxygenase 1 (HMOX1) and cytochrome P-450s (e.g., CYP1A1 involved in bioactivation of polyaromatic hydrocarbons) (Fig. 4). The differential gene expression patterns observed in the present study are consistent with previous studies of HBE cellular response to MSS^35^,^54^.

#### Differential gene expression following exposure to e-vapor lacking nicotine

One of the unknowns about e-cigarettes is the extent to which e-vapor alone without any additives influences cellular physiology or potentiates aphysiological behaviors in e-vapor exposed cells. To address this question we treated HBE cells with e-vapor without nicotine or other additives. EV0- treated cells were compared with the AT control cells and only a very small number of genes were found to be differentially activated following 1 h of exposure (Table 2). A total of 6 genes showed significantly altered expression in EV0 versus their corresponding AT control. Among these genes are RPS8 and ZNF721, genes that are involved in translation and transcription regulation (Supplementary Table S9). At 4 h post-exposure, four differentially expressed genes, including CYP1A1 one of cytochrome P-450s, can be detected (Supplementary Table S10). At 24 h post-exposure only two genes DEGs, CPEB1 and ZNF275, were related with gene expression. And another three DEGs, MUC5AC, SERPINA3 and TOP2A, were involved in response to wounding and stress (Supplementary Table S11).

**Figure 5:**
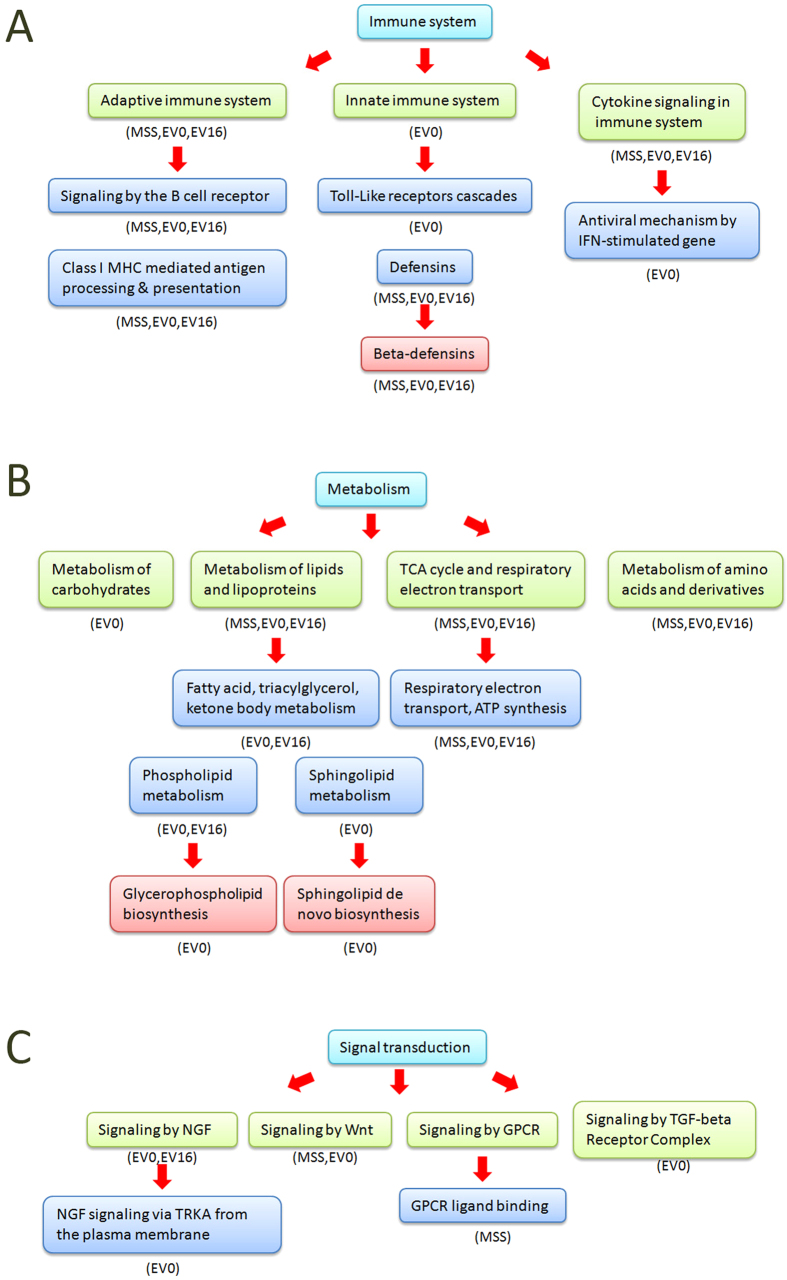
The significantly enriched pathways in GSEA results. Shown are the different pathways significantly enriched following exposure of HBE cells. AT, air treated control, MSS, mainstream smoke treated, EV0, e-vapor (0 mg/ml nicotine)-treated, EV16, e-vapor (16 mg/ml nicotine)-treated. Different colors represent different classes of subcategory. The name of the treatments appear below the pathways where enrichment was observed. (**A**) Category of immune system. (**B**) Category of metabolism. (**C**) Category of signal transduction.

Although few DEGs can be identified in EV0 treated samples at three different time points, GSEA identifies numerous pathways as being significantly enriched. A total of 56, 84 and 61 gene sets are significantly enriched at nominal p value < 0.05 in the EV0 treated cells at 1 h, 4 h, and 24 h, respectively, compared with the AT control (Supplementary Table S26). The significantly enriched biological pathways have diverse biological functions (Fig. 5 and Supplementary Tables S23–25) suggesting that e-vapor exposure alone has broad effects on basic cellular metabolism, transcription and translation and that this effect is persistent to 24 h after exposure. Not surprisingly we see that in all EV0 treatments categories for cell cycle associated functions (e.g., cell cycle checkpoint regulation, control of mitosis) are enriched as are a large number of categories involved in immune system function (Fig. 5A), suggesting that e-vapor alone can stimulate a prolonged response. Among the intriguing metabolism categories of gene expression enrichment showing major effects in all three EV0 treatments are those associated with the metabolism subcategory, lipids, phospholipids, and lipoproteins (Fig. 5B). Enrichment in the fatty acid triacylglycerol and glycerophosolipid biosynthesis pathways (the later especially in the EV0 24 h treatment; Supplementary Table S25) is particularly interesting since products of this pathway include glycerin and propylene glycol, the two major components in the e-vapor. To further investigate this response, we examined the expression level of the different individual genes in these pathways and discovered that many were significantly down-regulated as a result of e-vapor treatment (Supplementary Fig. S6). We interpret this observation as indicative that phospholipid and fatty acid triacylglycerol metabolism pathways play an import role in cellular response to e-vapor and as discussed below, this response is independent of whether or not nicotine is present since these genes are affected in both the EV0 and EV16 treatment samples.

#### Differential gene expression following exposure to e-vapor containing nicotine

E-vapor can be purchased containing a range of nicotine concentrations, with the expectation that over time decreasing the amount of added nicotine in the e-vapor could help wean the user from nicotine dependence and contribute to smoking cessation. In our study we were particularly interested in determining if a cellular signature on transcription is created by the presence of nicotine in the e-vapor. Therefore, we compared differential gene expression in HBE cells exposed to e-vapor generated from e-liquid lacking (EV0) or containing (EV16) 16 mg/ml nicotine.

Following 1 h of exposure many more DEGs can be detected in EV16 exposed HBE cells (EV16 1 h) than the AT (control) cells (Table 2 and Supplementary Table S6). Fewer DEGs are observed 4 h and 24 h after exposure ceases (EV16 4 h and EV16 24 h) and these cell samples show expression profiles similar to what was observed in the EV0 (no nicotine) treated cells (Table 2 and Supplementary Tables S7 and S8). As a consequence functional annotation cluster analysis could only be carried out on the DEGs at the 1 h time point (EV16 1 h). However, GSEA was applied for the gene sets enrichment analysis at three different time points.

In EV16 1 h samples, 57 genes showed significant differential expression relative to AT 1 h controls and of these 43 genes were down-regulated (Table 2). The significantly regulated genes were enriched in different functional clusters, including cell cycle, response to hypoxia, response to organic substance, apoptosis and acute inflammatory response clusters (Supplementary Table S15). The high proportion of DEGs exhibiting down-regulation likely signifies that e-vapor with nicotine elicits an immediate toxic response in HBE cells. Among the processes negatively regulated in HBE cells at this time point in contrast to cells exposed to EV0 were processes associate with cellular transcription/transcription factor function (Supplementary Table S15) suggesting that the presence of nicotine had a broad suppressive effect. Among the pathways significantly activated at this time point are cytochrome P450 function, retinoid metabolic process, and vitamin A metabolic process, cellular activities previously correlated with the metabolism of nicotine^55^.

At 4 h post-exposure, the number of DEGs sharply decreased compared with the 1 h exposure sample (Table 2). Among the DEGs are cytochrome P-450s CYP1A1 and CYP26A1 also identified in the 1 h treated culture and in the MSS treated samples (Supplementary Table S7). Genes involved in the MAPK signaling pathway, including MMP9, were also significantly differentially expressed (Supplementary Table S7), suggesting that the MAPK signaling pathway was still be significantly activated at this time point. After analysis with DAVID, the annotation clusters related with defense response and regulation of cell proliferation were significantly enriched compared with the air control (Supplementary Table S16). Although slightly fewer enriched clusters can be identified in EV16 4 h compared to 1 hr samples, cluster responded to wounding still can be detected which indicated that the HBE cells were still affected by e-cigarette vapor with 16 mg nicotine but recovered from the influence of Ev16 compared with 1 h exposure samples. At 24 h post-exposure, nine genes, including OASL (related with immune response), TOP2A and TOP2B (both of the genes related with DNA metabolic process) were differentially expressed in HBE cells (Supplementary Table S8). DAVID analysis of the DEGs cannot detect any significant enriched annotation cluster.

It was possible to identify significantly enriched gene sets in EV16 treated HBE cells relative to AT control cells at all treatment time points using a nominal P value < 5%. At 1 h, 4 h and 24 h a total of 43, 36 and 40 gene sets were identified (Supplementary Table S26). The enriched gene sets represents a number of pathways with different biological functions, and indicates that the effects of e- vapor containing nicotine on HBE DNA replication, cell cycle control, cellular transcription, translation, and metabolism (Supplementary Tables S20–22) persists after the initial exposure to the aerosol has stopped.

#### Comparison of gene expression in HBE cells exposed to e-vapor versus MSS

A total of 18 genes were identified whose differential activation overlapped between MSS and EV16 treated HBE cells. Seven (7) of these genes show identical patterns of expression with the majority (i.e., ZNF275, TOP2B, NR4A1, DDIT4, CYP26A1, SIK1) being significantly down-regulated in both MSS and EV16 treated cultures. This finding may be related to the presence of nicotine. The results of enrichment analysis indicate that the overlapping genes are involved in regulation of cell cycle and regulation of transcription. CYP1A1 was up regulated in all three different treated cultures. In contrast, zinc finger protein 275 (ZNF275) were significantly down regulated in MSS, Ev16 and EV0 treated samples.

A large part of the DEGs (91%) were down regulated in EV16 treated cultures after 1 h exposure. On the contrary, more (67%) up regulated DEGs were identified in MSS treated sample at the same time point. The percentage of up regulated genes were much higher at 4 h and 24 h time points, almost arrived 95%. In contrast, few DEGs can be discovered in EV16 and EV0 treated samples at these two points.

Many significantly regulated pathways overlap between the MSS and EV16 treated HBE cell cultures after 1 h and 4 h exposure. Included among these are pathways for response to hypoxia, regulation of cell cycle, response to organic substance, inflammatory response and immune responses. This result indicates that e-vapor with nicotine affects HBE cells in the same manner as exposure to MSS in the time points immediately following exposure. Pathways discretely enriched can also be found in both EV16 and MSS treated samples. In MSS treated cells the two uniquely activated pathways are those associated with basal metabolic processes, namely positive regulation of nitrogen compound metabolism and positive regulation of macromolecule biosynthesis. In EV16 treated cell cultures, the vitamin A and retinoid metabolic pathways are uniquely activated.

The results of GSEA were also consistent with what we discovered previously. Pathways involved in cell cycle and response to stress were enriched in both MSS and E-vapor treated samples. We further compared MSS and E-vapor treated cultures in three main functional categories (Fig. 5), including immune system, metabolism and signal transduction. Three subcategories of immune system were enriched in different treated cultures. Adaptive immune system and cytokine signaling in immune system were enriched in all the samples (MSS, EV0, EV16). Although innate immune system were only enriched in EV0 treated samples, β-defensins, the subset of it were also identified significantly regulated in different treated samples(Fig. 5A), which indicated both E-vapor and MSS will influence the immune system in some similar aspects. Most subcategories of metabolism were also enriched in all the treated samples (Fig. 5B). Nevertheless, the subset of lipids and lipoproteins, including fatty acid, triacylglycerol, ketone body metabolism, phospholipid metabolism and sphingolipid metabolism, were can only be discovered enriched in e-vapor (EV16, EV0) treated cultures (Fig. 5B) which inferred that these pathways were influence by the compounds only in E-vapor, like glycerin and propylene glycol. In the category of signaling transduction, components of the *Wnt* and GCPR signaling pathways were enriched in both MSS and e-vapor treated cultures. In contrast, signaling by Nerve Growth Factor (NGF) and Transforming Growth Factor-β (TGF-β) receptor complex were only enriched in e-vapor treated samples (Fig. 5C). NGF is known to have a modulatory role in the immune system and in the regulation of specific neuroendocrine associated and is elevated with severe stress and following smoking cessation^56^. Moreover, Wongtrakool *et al*.^57^ have reported that nicotine stimulates NGF release by lung fibroblasts through α7 nicotinic acetylcholine receptor (α7 nAChR)- and nuclear factor-κB (NFκB)-dependent pathways suggesting that this may contribute to tobacco smoke-induced airway hyper-responsiveness found in asthma. TGF-β1 on the other hand has been suggested to be involved in the development of COPD and individuals with COPD and cigarette smokers exhibit increased levels of TGF-β1 signaling^58^.

## Discussion

In this study, we evaluated whether exposure of HBE cells to e-cigarette aerosols (e-vapor) has a noticeable effect of HBE cellular functions and transcriptional activities and if the nicotine present in e-vapor has a recognizable cellular response signal. We also compared the effects of exposure e-vapor to that of exposure to MSS from conventional tobacco cigarettes, a well-documented elicitor of cellular response and contributing factor to the development of COPD and lung cancers. Our goal was to determine whether e-vapor poses a risk to the human airway epithelium. At the present time, data on the effects of e-vapors on human cellular function are limited and most studies have not made direct comparisons with tobacco smoke. Our results indicate that while e-vapor does not elicit many of the cell toxicity responses observed in MSS-exposed HBE cells, e-vapor exposure is not benign but elicits discrete transcriptomic signatures with and without added nicotine.

### Understanding the differential effects of e-vapor and MSS

Maunders *et al*.^35^ previously reported that HBE cells exposed to either filtered air or diluted MSS resulted in changes in the transcriptome that could be observed by 1 h post exposure. These changes included decreased expression of cellular adhesion genes, increased intracellular permeability, and increased expression of antioxidant and detoxification genes. By 24 h post-exposure, the deactivated expression of genes controlling general cellular function was restored to pre-exposure levels. However, genes involved in cell cycle control and apoptosis continued to increased. Using both DAVID and GSE analysis to characterize transcriptomic changes we found that all three MMS treatments (ST 1 h, ST 4 h, and ST 24 h and two of the e-vapor treatments (EV16 1 h and EV16 4 h) were capable of eliciting significant changes in the HBE cell transcriptome. As might have been expected based on studies preceding this work, MMS exposed HBE cells showed significantly robust responses. In these treatments, a large number of the significantly regulated gene enriched pathways were associated with signal transduction annotation clusters, such as MAPK signaling pathway, cell cycle regulation, apoptosis, response to organic substances and response to hypoxia. These data suggest that the MAPK signaling and the downstream pathways were activated after the exposure of MSS and nicotine containing e-vapor. After the activation of the MAPK signaling pathway, downstream pathways including alterations in cell cycle and apoptosis were also activated. HBE cells also exhibited up-regulation in pathways associate with response to organic substance and hypoxia (Fig. 2). The significantly regulated pathways cluster according DAVID analysis in several main groupings: (1) MAPK signaling pathway (ST 4 h, ST 24 h), (2) Cell cycle cluster (ST 1 h, ST 4 h, ST 24 h, EV16 1 h), (3) Apoptosis cluster (ST 1 h, ST 4 h, ST 24 h, EV16 1 h), (4) Response to organic substance cluster (ST 1 h, ST 4 h, ST 24 h, EV16 1 h) and (5) Response to hypoxia cluster (ST 1 h, ST 4 h, ST 24 h, EV16 1 h). Our findings are consistent with the earlier reports^35^ who noted the importance of the MAPK signaling pathway. The decrease expression of cell adhesion genes in response to smoking has been suggested to be part of a synergistic interaction between smoke components and factors contributing to allergen activated inflammatory responses^59^,^60^,^61^. Our studies also uncovered alterations similar to those reported from work involving mouse models of MSS exposure^62^,^63^ where enhance expression of genes associated with inflammatory cell influx, activation of the NF-κB and p38 MAPK (mitogen-activated protein kinase) pathway genes were reported.

Both significantly up-regulated and down-regulated gene expression was found in MSS treated HBE cells beginning in the 1 h treatments and persisting throughout the 4 h to 24 hr recovery phase. More down regulation of expression was seen at 1 hr, whereas the majority of the significantly differentially expression genes were up-regulated at 4 h and 24 h time point. This clearly reflects the fact exposure to MSS is cytotoxic and therefore one expects that many biological pathways within the HBE cells would be shut down. After exposure, during cellular recovery, a large number of genes were significantly up-regulated and this activation persists through 24 h post MMS exposure (ST 24 h).

Fewer significantly regulated genes can be identified in EV16 (e-vapor plus nicotine) treated cells indicating that the response to this insult is less dramatic than to smoke. The pattern of gene differential expression was also quite different in the EV16 treated HBE cells than that found in the MMS treated cells with a large part of the DEGs at 1 h being down-regulated and very few DEGs detected at 4 h and 24 h post e-vapor exposure. Those genes differentially expressed in EV16 treated cells 4 h post-exposure clustered in the response to wounding category (Supplementary Table S16), which suggests that the effects of EV16 exposure are maintained for a short time.

The differences observed in the transcriptomic profiles between MSS and EV16 can be attributed to the dramatic difference in composition of MSS and e-vapor. In EV16 only one compound, namely nicotine, has been implicated as cytotoxic to human cells. The general response to EV0 in HBE cells (i.e., down-regulation of gene expression for basic cellular functions) is also seen in EV16 cell, and the relatively few DEGs identified at 4 h and 24 h post exposure (Table 2 and Supplementary Tables S7 and S8) likely constitute the nicotine-specific aspects of the response. Indeed this is where differences are most readily seen between EV0 and EV16 treated HBE cells.

Our results suggest that while e-vapor containing nicotine has substantially less effects than MSS and its effects are less persistent than those of smoke exposed cells, exposure to e-vapor alone and e-vapor containing nicotine is not without cellular consequence. As noted above, although EV0 shows relatively small effects on the human HBE cell transcriptome, those genes affected are grouped by GSEA into the same pathways affected in EV16 treated cells at the various time points (Supplementary Tables S23–25).

Interestingly, both the phospholipid and fatty acid triacylglycerol metabolism pathways were enriched in all of the five e-vapor treated cultures (EV0 1 h, 4 h, 24 h; and EV16 4 h, 24 h), but these two pathways did not show enriched in any of the MSS-treated HBE cell samples (Fig. 5B and Supplementary Tables S17–25). Moreover, the glycerophophlipid biosynthesis pathway, the subpart of phospholipid metabolism pathway, also can be detected enriched at Ev0 treated culture at 24 h time points (Supplementary Table S25). Two main chemical compounds, glycerin and phosphatidate, were suggested to be involved in the glycerophospholipid biosynthesis pathway. Based on this finding, we propose that the exposure to glycerin, a main ingredient of e- vapor, alters cellular phospholipid and fatty acid triacylglycerol metabolism and in particular glycerophopholipid biosynthesis.

**Figure 6:**
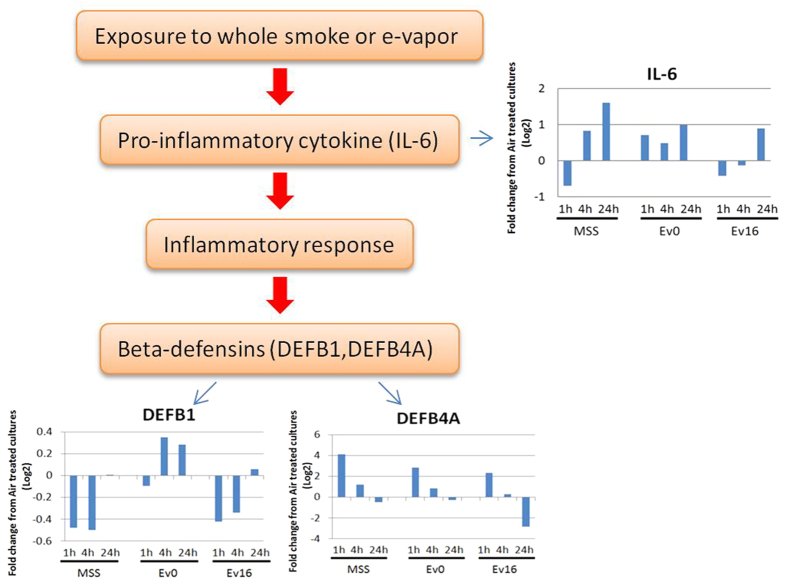
The inflammatory response influenced by e-vapor and MSS in HBE cells. Shown are the genes involved in the inflammatory response influenced by e-vapor and main stream smoke in HBE cells. The fold change of genes expression level from air treated cultures in different treated samples at three time points were shown in the bar plots.

In previous studies, innate immune defense against respiratory viral infection were shown to be affected by the e-vapor^45^. In our study, we similarly observed that both MSS and e-vapor significantly affect gene expression associated with three subcategories related to immune function: adaptive immune system, innate immune system and cytokine signaling in immune system (Fig. 5A). Two independent studies^45^,^64^ have reported that HBE cells expressed higher levels of IL-6 following exposure to e-vapor with or without nicotine. We noted a similar phenomenon in our studies, observing that the expression of IL-6 was up-regulated in HBE cells exposed to EV0 at all time points (Fig. 6). In contrast to these earlier studies we found that 1 h exposure to either EV16 or MSS resulted in the down-regulation of IL-6. The reason for the difference among these results is not known and whether this is related to the absolute amounts of nicotine or derivatives delivered will require further investigation.

β-defensin is an antibiotic peptide which is locally regulated by inflammation. GSEA found that the β-defensin pathway was significantly enriched [in both EV0 and EV16 treated] HBE cells treated by e-vapor [and MMS] suggesting that β–defensin is a downstream regulated pathway following inflammatory response resulting from exposure to e-vapor (Fig. 6). Only two β-defensin genes, DEFB1 and DEFB4A, were found to be expressed in HBE cells following any of the treatments. DEFB4A was significantly up-regulated in EV0, EV16 and MSS treated HBE cells at 1 h. In contrast, DEFB1 was [not affected or up regulated] in HBE cells following e-vapor treatments lacking nicotine (EV0 1 h, EV0 4 h), but down-regulated in HBE cells exposed to MSS and EV16 at 1 h and 4 h (ST 1 h and ST 4 h; EV16 1 h and EV16 4 h). This might reflect some inhibitory effect of the presence of nicotine or a derivative in MSS.

How alterations in cellular transcriptomic profiles observed *in vitro* compares to what occurs in these tissues *in vivo* following smoke exposure is not fully understood. Spira *et al*.^27^ were among the earliest investigators to analyze global gene expression in the lungs of smokers and non-smokers. Using direct collection of HBE cells from main-stem bronchi in healthy never-smokers these investigators were able to describe the nature of gene expression in what they characterized as normal-appearing epithelial cells and contrast that with global expression in smoking exposed HBE cells. They reported that the majority of genes expressed in normal cells are protective genes, antioxidant- and phase 1 and 2 detoxification drug-metabolizing genes, and a number of ion-transporting genes. Expression of 361 genes displayed highly significant differences in their level of expression between never and current smokers. Spivack *et al*.^65^ used real-time polymerase chain reaction (PCR) to show that the expression of a number of carcinogen- and oxidant metabolizing genes was altered in buccal mucosal cells of smokers, although there was substantial variability in gene expression between subjects. These researchers later showed that expression of a number of antioxidant- and phase 1 and 2–metabolizing genes was altered in both buccal mucosal and bronchial epithelial cells from smokers^66^. Subsequent studies by Sridhar *et al*.^67^ found similar evidence of differential activation of gene expression from an examination of the nasal, oral, and bronchial epithelial cells of smokers and nonsmokers further indicating that smoke exposure has significant persistent effects on cellular transcription not observed in never or non-smokers.

Gene expression alterations similar to those observed in the lung/bronchia have also been reported to occur in the epithelial cells of the nasal and oral tissues of smokers^10^,^67^,^68^. However, because these studies utilized different treatments (e.g., whole smoke, smoke extracts or other condensate) and were conducted using different immortalized or transformed cell lines, making a direct comparison among levels of gene activation/deactivation and pathway enrichment is difficult. However, in general, patterns of gene expression associated with decreased expression of cellular adhesion genes, increased intracellular permeability, and increased expression of antioxidant and detoxification genes are observed.

Beane *et al*.^69^ examined large airway epithelial cell RNA from never smokers and current smokers with and without lung cancer using both microarrays and high-throughput sequencing. They observed that among the genes that were differentially expressed in smokers were members of pathways associated with metabolism of xenobiotics and retinol metabolism. They also found that the chemokine signaling pathways, cytokine-cytokine receptor interactions and cell adhesion molecules were differentially expressed in smokers with lung cancer. Hackett *et al*.^70^ subsequently showed that smoking was associated with a shift from Clara cells to mucous-secreting cell differentiation and that smokers compared to non-smokers showed enhanced expression of genes in secretory cellular differentiation, secretory mucosal defenses and mucociliary genes.

These comparative studies (smokers vs never or non-smokers; smokers with and without cancer) have also led to the development of gene expression–based biomarkers for the detection of lung cancers^28^,^70^,^71^. Recently, Schembri *et al*.^32^ showed that a large number of miRNAs are differentially expressed (mostly decreased) in bronchial epithelial cells of smokers and may directly alter expression of many airway epithelial cell genes in response to smoke. Altered miRNA expression has also been implicated as an early event in the progression of normal bronchial tissue to squamous cell lung cancer^72^. Whether e-vapor exposure results in altered miRNA levels similar to smoke remains to be determined.

This is one of first studies to apply transcriptome profiling to determine if e-vapor alone poses a risk to the human airway as well as to investigate the comparative risk posed by e-vapor exposure relative to that of MSS. We also explored whether transcriptomic profiling is useful in recognizing gene activation/deactivation signatures specifically associated with nicotine. Recognition of such responses would be useful in subsequent studies designed to parse out the cellular responses created by individual additives to e-liquid to simulate a smoke response or as flavorant. Our results indicate that while-e-vapor does not elicit many of the direct effects of MSS or smoke condensates previously observed both *in vitro* and *in vivo* studies of cellular response, e-vapor exposure is not benign and both vapor with and without added nicotine have definable transcriptomic effects. The observed significantly enriched pathway, including the phospholipid and fatty acid triacylglycerol metabolism pathway, at different time points of e-vapor treated cultures demonstrate the effects and also suggest a possible mechanism of how the e- vapor affects HBE cells. In addition, the data clear show that different effects on the HBE transcriptome occur in response to the presence of nicotine.

Our findings are in contrast to a recent report from Misra *et al*.^64^ who concluded from an analysis of exposure to e-cigarette liquids and aerosols across several doses up to 100-fold higher than typical smoke exposure had little or no effect on cellular toxicity, levels of cytokine IL-8 response or other inflammatory markers measured when compared to tobacco smoke. They also reported no difference in the cytotoxic, genotoxic or inflammatory response of cells to e-liquid and aerosol between e- vapor alone and vapor containing nicotine. While the general conclusion of their work suggests that e-cigarettes have decreased human health impacts compared to conventional tobacco cigarettes, our finding support the notion that much more caution needs to be applied prior to endorsing these products as risk free alternative. Indeed, our data would suggest that the more complex that composition of the e-liquid and vapor (due to the presence of alkaloid, additives, and flavorants) the more likely that significant cellular responses will be recognized. While the limitations of this study are that exposure measured was acute, we also do not know what the long term effect of e-cigarette use (e.g., vaping) is on the human airway, and if significant effects of persistent use will be recognized between “vapers” and “never-vapers” in a manner consistent with what has been found for smokers and never smokers. The fact that e-vapor containing nicotine affects signaling by NGF and others have shown that nicotine stimulated NGF release contributes to tobacco smoke-induced asthma raises significant questions about prolong use of e-cigarettes in this regard. These studies lay the groundwork for future analysis that might better inform the FDA and other governmental regulatory bodies in discussions of future regulation.

## Methods

### Growth of human bronchial cell cultures

Primary normal human bronchial epithelial (HBE) cells were purchased from Lonza Group Ltd (Walkersville, MD). The HBE cells were isolated from the epithelial lining of airways above the bifurcation of the lungs of healthy non-smoker 16 and 26 year old females, respectively (Catalog number CC2541; Donor 1: Tissue Acquisition Number 17784/Lot number 0000116705; Donor 2: Tissue Acquisition Number 16843/Lot number 0000072920). These cells were isolated from donated human tissue after obtaining permission for their use in research applications by informed consent or legal authorization. The cells are supplied for research use only. The HBE cells used in this study tested negative for bacterial, fungal, and mycoplasma contamination as well as were certified as testing negative for HIV-1, Hepatitis-B, and Hepatitis-C. All experiments conducted with these human-derived materials were performed in accordance with the relevant “Inventory and Activity Registration (IAR)” guidelines and regulations set forth by the University of Virginia Institutional Biosafety Committee (IBC) who reviewed and approved all experiments under IBC Number: 662-09. The protocol and experimental plan was approved throughout the duration of the experiments. All human-sourced materials were handled at the biological safety level 2 to minimize exposure of potentially infectious products, are required by the UVa IBC. All personnel involved in these studies were required to complete annual certification for Bloodborne Pathogen and Biosafety Training for Research Personnel.

Cell growth and differentiation protocols were adapted from Maunders *et al*.^35^ with minor modifications. Cells at passage 3 were seeded onto cell culture inserts (Transwell-Clear, 6.5-mm diameter, 0.4-μM pore size; Corning) at a density of 7.5 × 10^4^ cells per insert. Proper seeding concentrations within these cultures were determined empirically in order to maintain maximal viable cells in culture and allow triggering of differentiation and the production of a ciliated surface-layer of cells in suspended chambers^35^. Spectrophotometric quantification of cell proliferation and viability in cell populations during differentiation, and before and after air. MSS, and e-vapor exposures were measured at 450 nm/620 nm with the cell proliferation reagent WST-1 (Sigma-Aldrich) according to manufacturer’s protocol. Cell viabilities in all samples were maintained at >95% for all treatments.

### Transepithelial electrical resistance (TEER) assays

Cell health and integrity, monolayer confluence, and cellular adherence were monitored before and following exposure to air, MSS and e-vapor was determined using three inserts per exposure type at various time points and treatments by measuring transepithelial electrical resistance (TEER) using a MilliCell-ERS (Millipore; Billerica, MA) device. Assays were conducted coincident with exposure to 100 μl of UltraCULTURE serum-free medium (Lonza) to the apical side of the tissues on each insert.

### Histological and immunohistological analysis

Visual and histological inspection was used to confirm the presence of multilayered, apically-ciliated, differentiated cultures between 21-and 23 days post-induction. Histological examination of plastic embedded sections of cultured cells was carried out to confirm that cultures generated a pseudo-stratified mucociliary morphology containing 50–70% ciliated cells, about 25% goblet cells, and about 30% basal cells by day 21. The state of differentiation in cultures over time was assessed by immuno-staining of fixed cells with antiserum against β-tubulin and α-MUC5AC biomarkers for ciliated and goblet cells, respectively^73^. For goblet immunocytochemistry measurements, representative cultures of cells were trypsinized, cytospin slides prepared, and the cells treated with the murine monoclonal antibody 45M1 (Neomarkers, Fremont, CA) at 2 mg/ml, The percentage of ciliated, basal and goblet cells in the population was determined by counting the number of positively staining cells.

### Cellular exposure to air, MSS, and e-vapor

Tobacco reference cigarettes 1R5F were obtained from the University of Kentucky and maintained in sealed plastic bags at 6 °C. Prior to use, the 1R5F cigarettes were pre-conditioned by humidification at room temperature in a closed chamber containing a 76/24 (v/v) glycerin/water solution.

Twenty-three (23) day-old differentiated Human bronchial epithelial (HBE) cell cultures were placed into custom-designed exposure chambers (fabricated by Curbridge Engineering, Southhampton, UK) as described by Phillips *et al*.^54^ and the cells were exposed to either air or mainstream smoke (MSS) generated from 1R5F tobacco reference cigarettes using a Teague Enterprises TE-10 smoking machine (Teague Enterprises, Davis, CA). The machine was set according to the International Organization for Standardization with the following parameters: a 35-ml puff drawn over 2 sec every 1 min period; a 6 min cycle per cigarette (5 min of smoking, 1 min non-smoking) yielding ~10 cigarettes smoked per 1 h of treatment. During this time period only the initial 1/3rd to 3/8ths of the cigarette was consumed, providing for an even yield of smoke without the pyrolysis of the concentrated deposit of smoke residue near the filter-end that occurs as the cigarette nears complete combustion. The air/smoke mixture generated by the machine was further diluted with filtered room air in a 2 L chamber to yield a constant amount of Total Particulate Matter (TPM) and Total Suspended Particulate (TSP) to the chamber. TPM and TSP were quantified as described previously^54^.

Cell cultures were exposed for 1 H to diluted MSS (Smoke Treated = ST) or filtered air (Air Treated = AT) as control and experiments were performed in triplicate. The cell cultures and circulating tissue media were kept at 37 °C throughout the experiment.

For e-vapor exposure HBE cell cultures in exposure chambers were treated for 1 hr at 37 °C to e-vapor (Vapor Treated = VT) or to filtered air (Air Treated = AT), or maintained in the sterile hood in the incubator as a further non-treated control. E-cigarette vapor was generated using a commercially available product “MAGMA brand” marketed by Volcanoecigs.com [http://www.volcanoecigs.com/]. MAGMA brand was selected based upon its ability to be reloaded with e-liquid and the general reproducibility of performance over long exposure times. A puffer box capable of repetitively administering e- vapor exposure equivalent to the IOS standard 35-ml puffs drawn over 2 sec every 1 min utilized in MMS experiments (see above) was fabricated using high speed peristaltic pump in line with an attached pressure meter/flowmeter to approximate the same air flow as generated by the Teague-10 machine (Teague Enterprises; Woodland, CA). E-cigarettes refill cartridges (e-liquids) were purchased commercially from the e-cigarette manufacturer [Volcanoecigs.com; http://www.volcanoecigs.com/] and contain either 0 mg/ml or 16 mg/ml nicotine (as reported in the product details). The level of nicotine in the e-liquid used in these experiments (i.e., 16 mg/ml) allowed us to empirically determine equivalent to the cells to provide similar nicotine exposure rates at that achieved by MSS treatment.

Purity and composition of e-liquid for generation of e-vapor was confirmed using standardized analytical methods on a Shimadzu GC-MS QP2010Plus (Columbia, MD) as described in^74^. Clinically pure tobacco alkaloid compounds purchased from Toronto Research Chemicals (North York, Ontario, Canada) were used as standards. The methanol-dissolved standards include myosamine, nicotine, nornicotine, anatabine, nitroso-anabasine, and nitroso-nornicotine in order of column retention time. Additionally, our library database correctly identifies all of the compounds successfully.

### RNA isolation and RNA-seq analysis

Air-treated (AT) control, MSS treated (ST), and e-vapor (EV) treated cells were at the time points indicated, the cells were immediately suspended in Triazol reagent (InVitrogen, Gaithersburg, MD) and flash frozen in liquid nitrogen. The cells suspension was stored at −80 °C until use. Total RNA was isolated with a PureLink MiniKit (Ambion, Austin, TX) according the manufacturer’s protocol. After purification and quantification, 0.5 μg of total RNA from each triplicate experimental time-point was reverse transcribed with SABiosciences RT^2^ First Strand Kit (Frederick, MD) and tested by qRT-PCR for quality using several housekeeping genes (i.e., hypoxanthine phosphoribosyltransferase 1, ribosomal protein L13a, and glyceraldehyde-3-phosphate dehydrogenase, and minus β-actin) before being used for Illumina sequencing. Samples were sequenced at the UVa Genomic Facility (UVa School of Medicine) using one lane for each sample on an Illumina Genome GAIIx Analyzer using a 83 × 83 bp paired-end cycle protocol.

### Transcriptome (RNA-seq) data analysis

FASTQC^47^ was used for initial reads QC metrics (base quality distribution). Sickle^48^ was used to trim low-quality ends. RNA-seq reads were mapped to the reference genome (EnsEMBL, GRC37) using Tophat v2.0.0^49^. Cufflinks was used to transcript assemble after the alignment step. All the transcripts generated from the sequence samples were merged by the Cuffmerge. The output files were separately imported into Cuffdiff for further statistical analysis. Differential expressed genes (DEGs) were identified with a significance threshold of q value (false discovery rate) < 0.05. R software (3.1.3) was used for principal component analysis (PCA) to cluster and explore the relationship between different samples and aid in identifying outlier.

### Functional annotation analysis of the differentially expressed genes

Functional annotation of the differentially expressed genes (DEGs) from the different datasets (including mainstream smoke (MSS), e-vapor alone (EV0), e-vapor plus nicotine (EV16), and air treated (AT) control) was performed using the Database for Annotation, Visualization and Integrated Discovery (DAVID) analysis^75^. The DEGs were analyzed using DAVID Functional Annotation Tool version 6.7 and the Fisher exact test (maximum probability < 0.05) was as used to determine enrichment probability for those pathways.

In addition, Gene-set Enrichment Analysis (GSEA) v2.2.0^76^ was used to recognize specific functional pathways that were significantly enriched in different treatment samples, using all of the expressed genes rather than just DEGs. Predefined gene sets in Reactome database^77^ from MSigDB v. 5.0 were preprocessed to exclude sets with <15 and >500 genes and 1000 iterations were performed per analysis with a ratio of classes metric used to rank genes based upon their differential expression across groups. Gene sets with a nominal *P*-value < 0.05 were considered statistically significant. The figure of genes interaction was performed using the Reactome FI plugin of Cytoscape^78^.

## Additional Information

**How to cite this article**: Shen, Y. *et al*. Transcriptome sequencing reveals e-cigarette vapor and mainstream-smoke from tobacco cigarettes activate different gene expression profiles in human bronchial epithelial cells. *Sci. Rep*. **6**, 23984; doi: 10.1038/srep23984 (2016).

## Supplementary Material

Supplementary Information

Supplementary Table S1

Supplementary Table S2

Supplementary Table S3

Supplementary Table S4

## Figures and Tables

**Table 1:** Experimental design, transcriptome sequencing and quality control result.

Treatment	Donor	Time point(hour)	Number of reads	QC result
Air treatment (AT)	1	1	29, 951, 033	
		4	36, 563, 966	
		24	39, 897, 976	
	2	1	40, 734, 981	
		4	42, 785, 060	
		24	44, 336, 294	
E-vapor (EV0) 0 mg/ml nicotine	1	1	41, 151, 411	
		4	41, 577, 282	
		24	40, 545, 029	Failed
	2	1	39, 548, 678	
		4	33, 465, 346	
		24	44, 490, 185	
E-vapor (EV16) 16 mg/ml nicotine	1	1	33, 887, 312	Failed
		4	41, 802, 365	Failed
		24	39, 032, 577	Failed
	2	1	29, 152, 118	
		4	40, 885, 872	
		24	28, 394, 709	
MSS (ST) Kentucky 1R5F Reference	1	1	30, 140, 360	
		4	41, 345, 795	
		24	40, 980, 601	
	2	1	41, 391, 596	
		4	42, 510, 881	
		24	38, 913, 418	

Four treatments, including mainstream smoke (MSS), nicotine free e-cigarette vapor (E-vapor 0 mg nicotine), regular e-cigarette vapor (E-vapor 16 mg nicotine) and the air control (Air treatment), were exposed to HBE cells. Two donor cell lines from two healthy 16 and 26 year old females were used in these studies (Lines 0000116705 donor 1 and 0000072920 donor 2). Time point 1, 4, 24 means 1, 4 and 24 hour post-exposure, respectively. Four samples were the outliers which failed to pass the quality control (QC) and excluded in our following analysis.

**Table 2:** Numbers of differentially expressed genes (DEGs) in HBE cells exposed to MSS and e-vapor at different false discovery rates (FDRs).

Time points	FDR (%)	EV0 vs AT		EV16 vs AT		MSS vs. AT	
		Neg	Pos	Neg	Pos	Neg	Pos
1 h	5	2	4	43	4	16	33
	10	3	5	58	8	30	59
	20	4	6	95	15	46	98
4 h	5	2	2	7	3	3	58
	10	2	2	12	3	6	74
	20	7	5	16	3	11	94
24 h	5	6	1	8	1	3	88
	10	10	1	12	1	5	108
	20	13	1	19	1	11	136

Shown are the number of DEGs identified among the three exposure treatments. EV0, EV16, MSS and Air mean treatments of nicotine free e-vapor, e-vapor plus 16 mg nicotine, mainstream smoke and the air control, respectively. Neg and Pos represent down- and up-regulated, respectively. DEGs at three different FDRs (5, 10, 20%) for samples collected at the three time points (1 h, 4 h, 24 h) are displayed in the table.
