# Supplementary material for: Transcriptome sequencing reveals e-cigarette vapor and mainstream-smoke from tobacco cigarettes activate different gene expression profiles in human bronchial epithelial cells
Source: Sci Rep. 2016 Apr 4;6:23984. doi: 10.1038/srep23984 (PMC4819171; doi:10.1038/srep23984)

Transcriptome sequencing reveals e-cigarette vapor and mainstream-smoke from tobacco cigarettes activate different gene expression profiles in human bronchial epithelial cells

Yifei Shen[1], Michael J. Wolkowicz[2], Tatyana Kotova[2], Longjiang Fan[1] and Michael P. Timko[2,3] *

[1] Research Center for Air Pollution and Health and Institute of Crop Science, Zhejiang University, Hangzhou 310058, China

[2] Department of Biology and [3]Department of Public Health Sciences, University of Virginia, Charlottesville, VA 22903

*mpt9g@virginia.edu; telephone: +1 (434)-982-5817

SUPPLEMENTAL TABLES AND FIGURES

Supplementary Table S1- S2. Enriched annotation clusters in Air treated HBE cell cultures between different time points.

Supplementary Table S1 Enriched annotation clusters in 1 hour air treated culture compared with 4 hour air treated culture.

Supplementary Table S2 Enriched annotation clusters in 1 hour air treated culture compared with 24 hour air treated culture.

Supplementary Table S3 – S11. DEGs in MSS, EV0 and EV16- treated HBE cell cultures compared with air treated control at 1 h exposure and 4 h and 24 h recovery times.

Supplementary Table S3 DEGs in MSS treated cultures compared with air treated control after 1 hour exposure.

Supplementary Table S4 DEGs in MSS treated cultures compared with air treated control after 4 hour exposure.

Supplementary Table S5 DEGs in MSS treated cultures compared with air treated control after 24 hour exposure.

Supplementary Table S6 DEGs in Ev16 treated cultures compared with air treated control after 1 hour exposure.

Supplementary Table S7 DEGs in Ev16 treated cultures compared with air treated control after 4 hour exposure.

Supplementary Table S8 DEGs in Ev16 treated cultures compared with air treated control after 24 hour exposure.

Supplementary Table S9 DEGs in Ev0 treated cultures compared with air treated control after 1 hour exposure.

Supplementary Table S10 DEGs in Ev0 treated cultures compared with air treated control after 4 hour exposure.

Supplementary Table S11 DEGs in Ev0 treated cultures compared with air treated control after 24 hour exposure.

Supplementary Table S12 - S16. Enriched annotation clusters in MSS and EV16 treated HBE cell cultures compared with air treated control at 1 h exposure and 4 h and 24 h recovery times.

Supplementary Table S12 Enriched annotation clusters in MSS treated cultures compared with air treated control after 1 hour exposure.

Supplementary Table S13 Enriched annotation clusters in MSS treated cultures compared with air treated control after 4 hour exposure.

Supplementary Table S14 Enriched annotation clusters in MSS treated cultures compared with air treated control after 24 hour exposure.

Supplementary Table S15 Enriched annotation clusters in Ev16 treated cultures compared with air treated control after 1 hour exposure.

Supplementary Table S16 Enriched annotation clusters in Ev16 treated cultures compared with air treated control after 4 hour exposure.

Supplementary Table S17 - S25. Significant enriched pathways identified by in MSS, EV0 and EV16- treated HBE cell cultures compared with air treated control at 1 h exposure and 4 h and 24 h recovery times.

Supplementary Table S17 Significant enriched pathways in MSS treated cultures compared with air treated control after 1 hour exposure in GSEA.

Supplementary Table S18 Significant enriched pathways in MSS treated cultures compared with air treated control after 4 hour exposure in GSEA.

Supplementary Table S19 Significant enriched pathways in MSS treated cultures compared with air treated control after 24 hour exposure in GSEA.

Supplementary Table S20 Significant enriched pathways in Ev16 treated cultures compared with air treated control after 1 hour exposure in GSEA.

Supplementary Table S21 Significant enriched pathways in Ev16 treated cultures compared with air treated control after 4 hour exposure in GSEA.

Supplementary Table S22 Significant enriched pathways in Ev16 treated cultures compared with air treated control after 24 hour exposure in GSEA.

Supplementary Table S23 Significant enriched pathways in Ev0 treated cultures compared with

air treated control after 1 hour exposure in GSEA.

Supplementary Table S24 Significant enriched pathways in Ev0 treated cultures compared with air treated control after 4 hour exposure in GSEA.

Supplementary Table S25 Significant enriched pathways in Ev0 treated cultures compared with air treated control after 24 hour exposure in GSEA.

Supplementary Table S26. Number of significantly enriched pathways with a nominal *P*-value < 0.05 in GSEA analysis compared with air treated control at three time points.

	1h	4h	24h
MSS	59	67	58
EV0	74	103	78
EV16	43	36	40

Supplementary Table S27. The expression levels of the housekeeping genes in each sample, measured in FPKM. The housekeeping genes are taken from Eisenberg & Levanon[50].

		AIR		MSS		EV0		EV16	
	name	donor 1	donor 2	donor 1	donor 2	donor 1	donor 2	donor 1	donor 2
1h	LDHA	531.351	381.871	535.853	505.449	446.512	409.904	437.682	335.859
	PGK1	311.126	265.046	342.043	369.682	302.917	258.585	275.97	247.662
	RPS27A	461.791	478.083	558.593	653.495	443.598	416.398	404.588	448.768
	RPL19	575.968	595.193	734.077	862.718	533.227	561.594	398.262	621.282
	ARHGDIA	43.2843	48.3051	52.0921	49.3267	43.9217	48.1435	33.862	55.467
	RPS18	0	0	0	0	0	0	0	0
4h	LDHA	345.154	266.456	372.052	360.463	339.027	340.939	317.749	287.438
	PGK1	276.355	237.463	273.087	307.553	279.746	260.774	291.47	243.323
	RPS27A	511.698	455.098	537.787	567.646	549.409	460.492	522.656	437.535
	RPL19	602.403	534.072	616.024	758.28	619.86	620.677	628.295	569.594
	ARHGDIA	54.7439	45.9909	52.8786	77.7861	55.6186	68.1928	46.7322	52.6485
	RPS18	0	0	0	0	0	0	0	0
24h	LDHA	471.435	303.227	437.039	411.153	402.172	323.631	340.618	363.148
	PGK1	275.101	262.773	334.295	288.716	291.819	255.491	271.779	223.31
	RPS27A	607.967	465.342	699.568	609.022	508.556	528.192	463.812	422.013
	RPL19	530.243	682.493	687.468	750.771	549.823	691.74	518.534	686.04
	ARHGDIA	42.4213	64.3935	68.7027	44.3547	46.6689	55.1103	57.9421	56.0166
	RPS18	0	0	0	0	0	0	0	0

Supplementary Figure S1. Histological and immunohistological examination of differentiated HBE cells in air-liquid interface cultures.

Supplementary Figure S2. Principal Coordinate Analysis (PCA) of samples after elimination of outliers.

Supplementary Figure S3. Comparison of gene expression in two matched donors.

Supplementary Figure S4. The number of differentially regulated genes found at a 5% FDR between different time points in air treated control samples.

Supplementary Figure S5. The number of differentially regulated genes found at a 5% FDR among the various treatments and time-points.

Supplementary Figure S6. The expression of the genes involved in the fatty acid triacylglycrol metabolism pathway in e-vapor treated HBE cells at 24 h.

Supplementary Figure S1. Histological and immunohistological examination of differentiated HBE cells in air-liquid interface cultures. Panel A. Upper photograph shows a plastic embedded section through multilayered, apically-ciliated, differentiated HBE cell layer growing in air liquid interface culture at 21-and 23 days post-induction. Note that basal cell, ciliated cells, and goblet cells are readily visible. Lower photographs show the results of immunohistochemical staining of comparable differentiated cell cultures viewed under confocal microscopy (left-cross section through monolayer; right - longitudinal section through monolayer) in which the basal cells are stained with DAPI (blue), goblet cells (microvilli) are stained with Alexa 658 mouse anti-MUC5AC antibody (red); and ciliated cells are stained with Alexa 488 rabbit anti β-tubulin (green). Panel B illustrates the time course of Transepithelial electrical resistance (TEER) during culture differentiation illustrative of the development of cellular confluence and strong cellular adhesion.

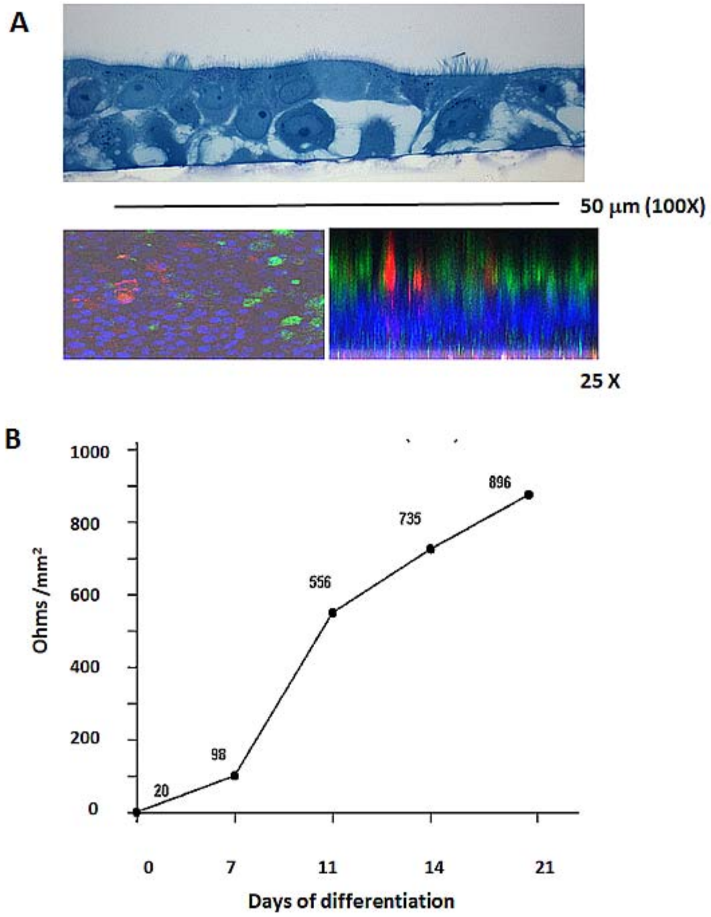

Supplementary Figure S2. Principal Coordinate Analysis (PCA) of samples after elimination of outliers. Shown are the results of PCA following the exclusion of samples deemed to be outliers. The results show that the samples are divided into two clusters, each cluster represents a donor in the experiment (two donors were termed by *_0 and *_1, respectively).

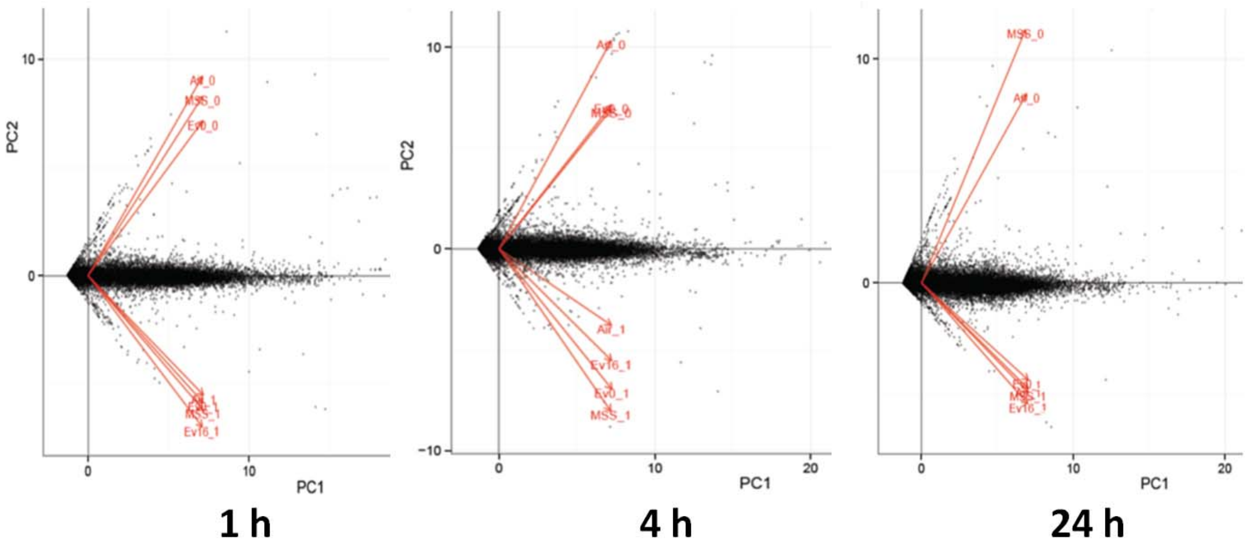

Supplementary Figure S3. Comparison of gene expression in two matched donors. Shown is the comparison of RNA-seq determinations of gene expression in two matched donors for the various genes after exposure treatment by air, for three different times (1 h, 4 h, 24 h). Expression was measured as fragments per kilobase of exon per million mapped sequence reads (FPKM); R2=0.94 (1 hour); R2=0.94 (4 hour); R2=0.91 (24 hour).

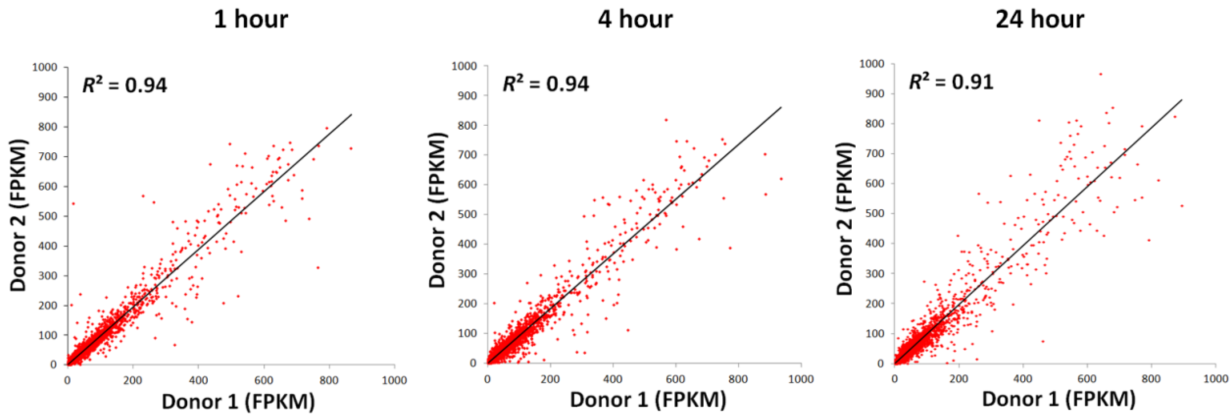

Supplementary Figure S4. The number of differentially regulated genes found at a 5% FDR between different time points in air treated control samples. Shown are the number of genes observed to be significantly differentially regulated for each pairwise interaction in air-treated control samples.

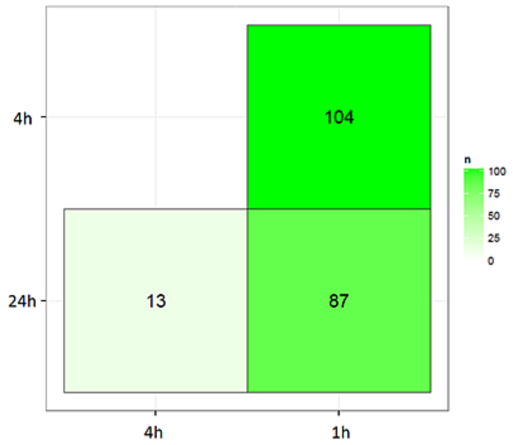

Supplementary Figure S5. The number of differentially regulated genes found at a 5% FDR among the various treatments and time-points. Shown are the number of genes observed to be significantly differentially regulated for each pairwise interaction tested of different treatment conditions and time points analyzed.

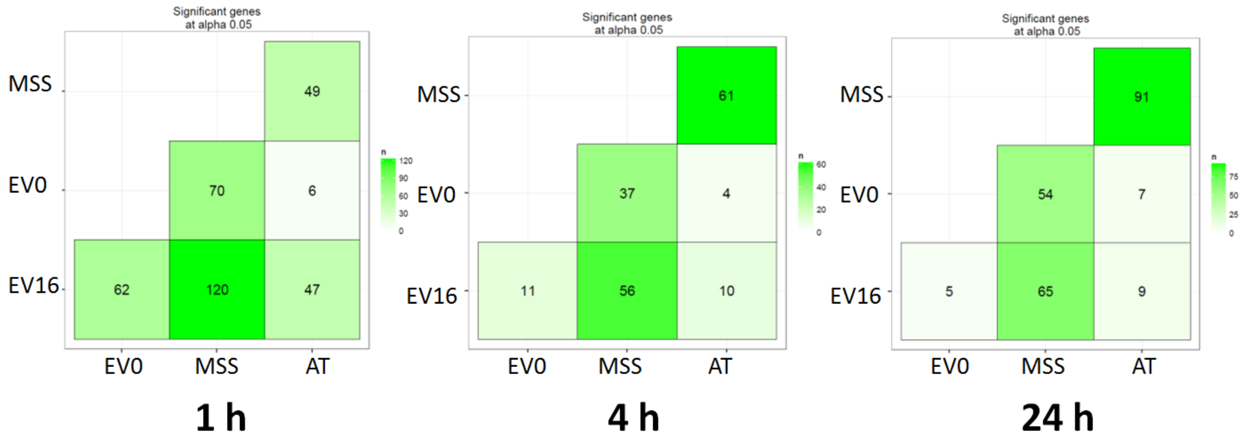

Supplementary Figure S6. The expression of the genes involved in the fatty acid triacylglycerol metabolism pathway in e-vapor treated HBE cells at 24 h.

Shown are the fold-change levels in expression for genes involved in the fatty acid triacylglycerol metabolism pathway in HBE cells exposed to EV0 (e-vapor containing 0 mg/ml nicotine). Fold-change level was calculated by EV0/(AT)-1 h if genes were up-regulated or -(AT)/(EV0)+1 if genes were down-regulated. Levels are indicated on blue-white-red color scale with the interval of the fold change level being ±1.5. Genes colored in green did not have detectable levels of expression. A large part of genes involved in the pathways were down-regulated compared with the AT control.

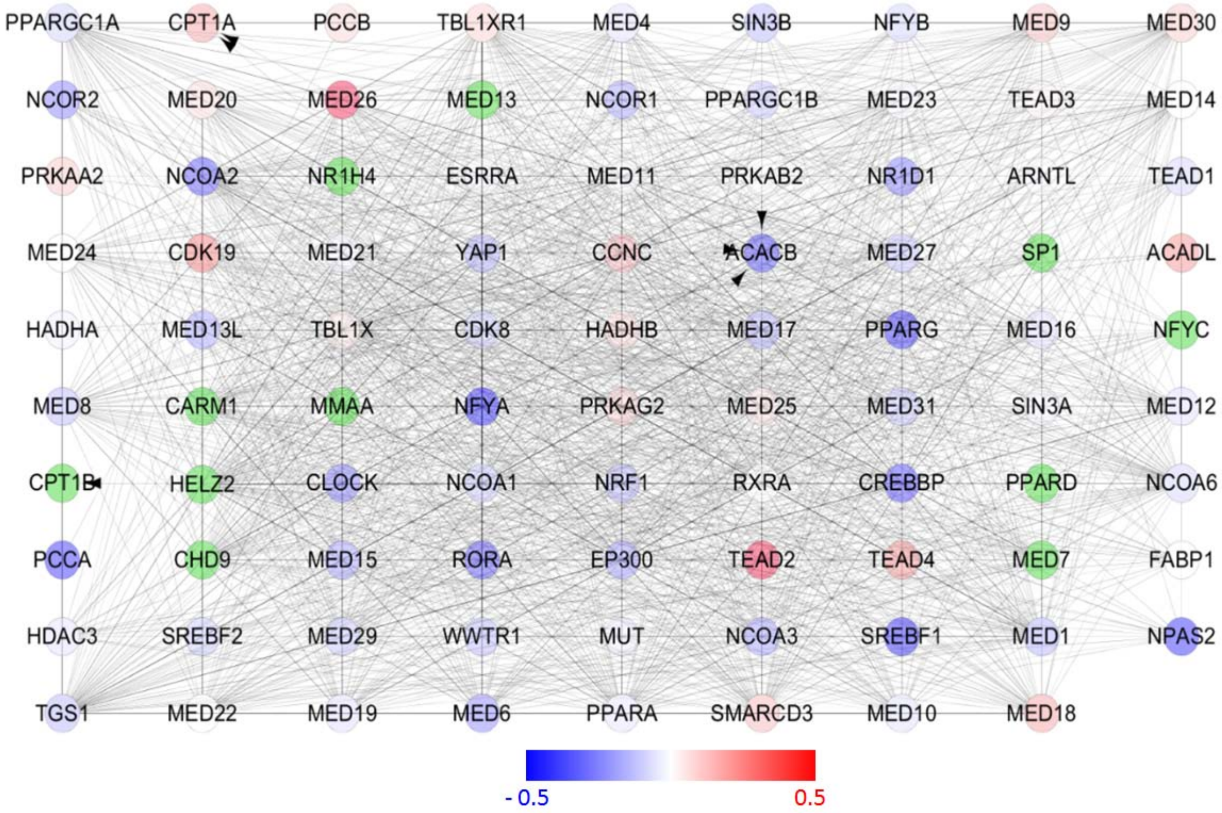

Supplement: Supplementary Information [file srep23984-s1.pdf]
